# 

**Published:** 1856-02

**Authors:** 


### RECEIPTS ON SUBSCRIPTIONS,

FROM JAN. 10TH, TO FEB. 15TH, 1856.

### ILLINOIS.

| | | |
|---|---|---|
| Dr. G. P. Ransom, | Vol. 4 & 5, | $4 00 |
| " H. K. Lathy, | Vol. 4, | 2 00 |
| " G. H. Young, | Vol. 4 & 5, | 5 00 |
| " M. C. Archer, | Vol. 5, | 2 00 |
| " L. Clark, | " 5, | 2 00 |
| " J. Blount, | " 5, | 2 00 |
| " J. A. Brenneman, | " 4, | 2 00 |
| " James Leeder, | " 4, | 2 00 |
| " E. Richards, | " 5, | 2 00 |
| " C. Goodhake, | " 5, | 2 00 |
| " W. W. Adams, | " 5, | 2 00 |
| " A. S. Haskell, | " 4, | 2 00 |
| " D. O. McCord, | " 4, | 2 00 |
| " O. S. Sohnson, | " 4, | 2 00 |
| " A. P. Finch, | " 5, | 2 00 |
| " R. M. Humble, | " 5, | 2 00 |
| " J. W. McKenny, | " 5, | 2 00 |
| " J. O. Long, | " 5, | 2 00 |
| " A. W. Heise, | Vol. 4 & 5, | 5 00 |
| " John Grinsted, | Vol. 5, | 2 00 |
| " J. H. McDill, | " 5, | 2 00 |
| " F. K. Bailey, | " 4, | 2 00 |
| " M. S. Hubbard, | " 4, | 2 00 |
| " J. C. Lowrie, | " 5, | 2 00 |
| " —— Jossoy, | " 4, | 2 00 |
| " R. B. Ray, | " 5, | 2 00 |
| " D. W. Stormont, | Vol. 4 & 5, | 4 00 |
| " J. W. Steele, | Vol. 5, | 2 00 |
| " N. P. Naramore, | " 4, | 2 00 |
| " M. H. L. Schooley, | " 4 & 5, | 5 00 |
| " A. Berchleman, | " 4 & 5, | 4 00 |
| " Jesse Barlre, | Vol. 4, | 2 00 |
| " G. N. Higgins, | " 5, | 2 00 |
| " Lee Smith, | Vol, 4 & 5, | 4 00 |
| " W. H Studley, | Vol. 5, | 2 00 |
| " Orrin Peak, | " 5, | 2 00 |
| " L. D. Martin. | " 4, | 2 00 |
| " D. Hess, | Vol. 4 & 5, | 4 00 |
| " H. C. McPherson, | Vol. 5, | 2 00 |
| " P. G. Corkins, | " 4, | 2 00 |
| " C. W. Knatt, | " 4, | 2 00 |
| " H. Vanmeter, | " 5, | 2 00 |
| Dr. H. Truesdail, | " 4, | $2 00 |
| " F. Erhard, | " 4, | 2 00 |
| " J. P. Walker, | " 5, | 2 00 |
| " S. R. Hyslap, | Vol. 3 & 4, | 5 00 |
| " James McCay, | Vol. 4, | 2 00 |

### INDIANA.

| | | |
|---|---|---|
| Dr. Wm. Manson, | Vol. 5, | $2 00 |
| " R. O. Wilson, | " 5, | 2 00 |
| " M. G. Mullinix, | " 4, | 2 00 |
| " R. C. Pierce, | " 4, | 2 00 |
| " S. T. Langdon, | " 5, | 2 00 |
| " J. P. Tucker, | Vol. 4 & 5, | 4 00 |
| " S. W. Ritchey, | Vol. 4, | 2 00 |
| " D. M. Marshall, | " 5, | 2 00 |
| " Jas. Emmons, | " 5, | 2 00 |
| " J. A. W. Berry, | " 5, | 2 00 |
| " C. Palmiter, | " 5, | 2 00 |
| " D. S. Caylor, | " 5, | 2 00 |
| " J. P. & T. A. Graham, | " 5, | 2 00 |
| " Jas. Evans, | " 5, | 2 00 |
| " G. A. Moss, | Vol. 3 & 4, | 4 00 |
| " Long & Ellis, | Vol. 5, | 2 00 |

### OHIO.

| | | |
|---|---|---|
| Dr. Thomas P. Bond, | Vol. 4, | $2 00 |

### IOWA.

| | | |
|---|---|---|
| Dr. J. W. Overhalts, | Vol. 5, | $2 00 |
| " H. Fisk, | " 5, | 2 00 |
| " J. H. Griffeth, | " 5, | 2 00 |
| " H. Clements, | Vol. 4 & 5, | 4 00 |
| " W. A. Daniel, | Vol. 5, | 2 00 |
| " W. Watson, | " 5, | 2 00 |
| " Thos. C. McGee, | " 5, | 2 00 |
| " J. D. Blanchard, | " 5, | 2 00 |
| " J. H. Bowers, | " 5, | 2 00 |
| " W. D. Nelson, | " 4, | 2 00 |
| " H. F. Brady, | " 4, | 2 00 |

### WISCONSIN.

| | | |
|---|---|---|
| Dr. O. Allen, | Vol. 5, | $2 00 |
| " H. L. Barnes, | " 5, | 2 00 |
| " C. W. Brigham, | " 4, | 2 00 |
| " Richard Jones, | " 4, | 2 00 |

### NEW YORK.

| | | |
|---|---|---|
| Dr. T. G. Cole, | Vol. 4, | $2 |

